# Retraction Note: Retinal degeneration driven by brain-derived neurotrophic factor deficiency in microglia and T-lymphocytes

**DOI:** 10.1038/s41598-026-62205-y

**Published:** 2026-07-13

**Authors:** Sabrina Reinehr, Julia P. Zehge, Katharina Klöster, Maike Mueller, Hasan H. Hendek, Michael Sendtner, H. Burkhard Dick, Ralf Gold, Stephanie C. Joachim, Simon Faissner

**Affiliations:** 1 Experimental Eye Research Institute, University Eye Hospital, Ruhr-University Bochum, In der Schornau 23-25, 44892 Bochum, Germany; 2 Department of Neurology, Ruhr-University Bochum, St. Josef-Hospital, Gudrunstraße 56, 44791 Bochum, Germany; 3 Institute of Clinical Neurobiology, University Hospital Wuerzburg, Versbacher Straße 5, 97078 Wuerzburg, Germany

Retraction of: *Scientific Reports* 10.1038/s41598-025-21423-6, published online 13 October 2025

The Editors have retracted this Article.

After publication, concerns were raised about some of the figures. Specifically,

- In Figure 1, image for Peripheral/Control in panel A appears to overlap with image for Central/Control in panel F;
- In Figure 2, there appear to be cloned areas in the DAPI signal in the image for Nestin + DAPI/Control in panel P;
- Image for Vimentin + DAPI/LF in Figure 2J and Gephyrin + DAPI/CLF in Figure 5A appear to have overlapping DAPI signal;
- In Figure 3G, the representative Control, CLF, and LF images were incorrectly sourced from 3 month LF animals;
- In Figure 4, image for Rhodopsin + DAPI/Control in panel A appears to overlap with image for Rhodopsin + DAPI/Control in panel D, and image for Rhodopsin + DAPI/CLF in panel A appears to overlap with image for Rhodopsin + DAPI/LF in the same panel.

The Authors confirmed the overlaps stating that the duplications, mislabelling, and panel swaps occurred during the figure assembly. The Editors therefore no longer have confidence in the data presented in this Article.

The Authors agree to the retraction but not to the retraction notice.

